# Photodynamic Diagnosis and Therapy for Peritoneal Carcinomatosis: Emerging Perspectives

**DOI:** 10.3390/cancers12092491

**Published:** 2020-09-03

**Authors:** Si Xu, Anne-Laure Bulin, Amandine Hurbin, Hélène Elleaume, Jean-Luc Coll, Mans Broekgaarden

**Affiliations:** 1 Institute for Advanced Biosciences, INSERM U1209, CNRS UMR5309, Université Grenoble-Alpes, 38700 La Tronche, France; sixu960805@sjtu.edu.cn (S.X.); Amandine.hurbin@univ-grenoble-alpes.fr (A.H.); mans.broekgaarden@univ-grenoble-alpes.fr (M.B.); 2 Department of Nuclear Medicine, Ruijin Hospital, Shanghai Jiao Tong University School of Medicine, Shanghai 200025, China; 3 Synchrotron Radiation for Biomedicine, UA07 INSERM, Université Grenoble-Alpes, European Synchrotron Radiation Facility, Biomedical Beamline, 38043 Grenoble CEDEX 9, France; anne-laure.bulin@inserm.fr (A.-L.B.); helene.elleaume@inserm.fr (H.E.)

**Keywords:** ovarian cancer, colorectal cancer, gastric cancer, pancreatic cancer, photochemotherapy, theranostic modalities

## Abstract

**Simple Summary:**

Peritoneal carcinomatosis, the formation of wide-spread metastases throughout the abdominal cavity, remains challenging to diagnose and treat. Photodynamic diagnosis and photodynamic therapy are promising approaches for the diagnosis and treatment of peritoneal carcinomatosis, which use photosensitizers for fluorescence detection or photochemical treatment of (micro) metastases. With the aim of highlighting the potential of this theranostic approach, this review outlines the clinical state of the art in the use of photodynamic diagnosis and therapy for peritoneal carcinomatosis, identifies the major challenges, and provides emerging perspectives from preclinical studies to address these challenges. We conclude that the development of novel illumination strategies and targeted photonanomedicines may aid in achieving more efficient cytoreductive surgery. In addition to combination treatments with chemo-, and radiotherapy, such approaches hold significant promise to improve the outlook of patients with peritoneal carcinomatosis.

**Abstract:**

Peritoneal carcinomatosis occurs frequently in patients with advanced stage gastrointestinal and gynecological cancers. The wide-spread peritoneal micrometastases indicate a poor outlook, as the tumors are difficult to diagnose and challenging to completely eradicate with cytoreductive surgery and chemotherapeutics. Photodynamic diagnosis (PDD) and therapy (PDT), modalities that use photosensitizers for fluorescence detection or photochemical treatment of cancer, are promising theranostic approaches for peritoneal carcinomatosis. This review discusses the leading clinical trials, identifies the major challenges, and presents potential solutions to advance the use of PDD and PDT for the treatment of peritoneal carcinomatosis. While PDD for fluorescence-guided surgery is practically feasible and has achieved clinical success, large randomized trials are required to better evaluate the survival benefits. Although PDT is feasible and combines well with clinically used chemotherapeutics, poor tumor specificity has been associated with severe morbidity. The major challenges for both modalities are to increase the tumor specificity of the photosensitizers, to efficiently treat peritoneal microtumors regardless of their phenotypes, and to improve the ability of the excitation light to reach the cancer tissues. Substantial progress has been achieved in (1) the development of targeted photosensitizers and nanocarriers to improve tumor selectivity, (2) the design of biomodulation strategies to reduce treatment heterogeneity, and (3) the development of novel light application strategies. The use of X-ray-activated PDT during whole abdomen radiotherapy may also be considered to overcome the limited tissue penetration of light. Integrated approaches that take advantage of PDD, cytoreductive surgery, chemotherapies, PDT, and potentially radiotherapy, are likely to achieve the most effective improvement in the management of peritoneal carcinomatosis.

## 1. Introduction

### 1.1. Peritoneal Carcinomatosis: Origins, Occurrence, Diagnosis and Treatment

Peritoneal carcinomatosis (PCAR) refers to the dissemination of cancer tissues throughout the peritoneal cavity. Although PCAR can be a primary form of cancer (peritoneal mesothelioma, primary peritoneal carcinoma and pseudomyxoma peritonei [1]), the majority of PCAR constitutes metastatic spreading from primary gastrointestinal and gynecological origins. PCAR with extra-abdominal origin accounts for only 10%, and the origins of 3–5% of PCAR remain unknown [2]. Due to the complexity of its origin, PCAR is not individually included in routine cancer statistics, making it hard to determine the total actual incidence and mortality rates. For example, colorectal cancer is the third common cancer, of which the peritoneum is the second most common metastatic site [3,4]. PCAR is identified in nearly 10% of colorectal cancer patients when initially diagnosed, and in 20–50% of patients with recurrent disease [3]. Similarly, up to half of the cases with recurrent gastric cancer develop peritoneal metastases [1]. Almost three-quarters of patients with ovarian cancer have wide-spread peritoneal dissemination [5], representing 46% of total cases of secondary PCAR [2]. Further, 9% of patients diagnosed with pancreatic cancer present synchronous peritoneal involvement [6]. Unfortunately, due to the lack of early diagnosis and curative therapy, the prognosis of PCAR is typically very poor [7,8].

PCAR is difficult to diagnose, as the clinical manifestation is atypical and also depends on the tumor’s origin. Although paraclinical tests and medical imaging, such as computed tomography, magnetic resonance imaging and ^18^F-deoxyglucose positron emission tomography, have diagnostic value, they lack sensitivity to detect minimal or microscopic metastases. In ovarian cancer specifically, occult peritoneal metastases can be observed in 4.2% of patients with early-stage disease [9]. PCAR are frequently discovered incidentally during surgical exploration [1,3]. 

In most cases, PCAR occurs in the absence of further systemic spread [1,3,10], which indicates that PCAR is a loco-regional disease [1,3]. This aspect makes it possible to provide local therapies, for which a promising clinical strategy is to combine cytoreductive surgery with hyperthermic intraperitoneal chemotherapy (HIPEC). Chemotherapeutics frequently given via HIPEC include oxaliplatin and mitomycin C, which can be further combined with synchronous intravenous agents such as 5-fluorouracil and folic acid [1,2,3]. Although several clinical trials have shown improved survival in highly selected patients [11,12,13], the effectiveness of the combination of cytoreductive surgery and HIPEC remains controversial due to a lack of high-grade evidence from randomized clinical trials. For example, the UNICANCER PRODIGE 7 trial presented that the addition of oxaliplatin-based HIPEC to cytoreductive surgery influenced neither overall survival nor relapse-free survival, and led to a high associated morbidity [14,15]. As such, the cytoreductive surgery + HIPEC combination is still not a definitive option recommended by most guidelines. In addition, cytoreductive surgery is frequently not extensive enough, as micrometastases are often missed during the procedure. In advanced ovarian cancer alone, the microscopic micrometastases can be detected in >20% of the patients [16,17]. Altogether, these factors necessitate new treatment strategies for the management of PCAR.

### 1.2. Photodynamic Diagnosis (PDD) and Photodynamic Therapy (PDT)

Over the years, photodynamic diagnosis (PDD) and therapy (PDT) have emerged as promising diagnostic and therapeutic procedures for PCAR. Both modalities utilize a common theranostic principle that involves the use of specific dyes (photosensitizers) and their excitation by visible and near-infrared light. Moreover, the modalities can, in principle, be easily combined into a single theranostic regimen.

**Figure 1:**
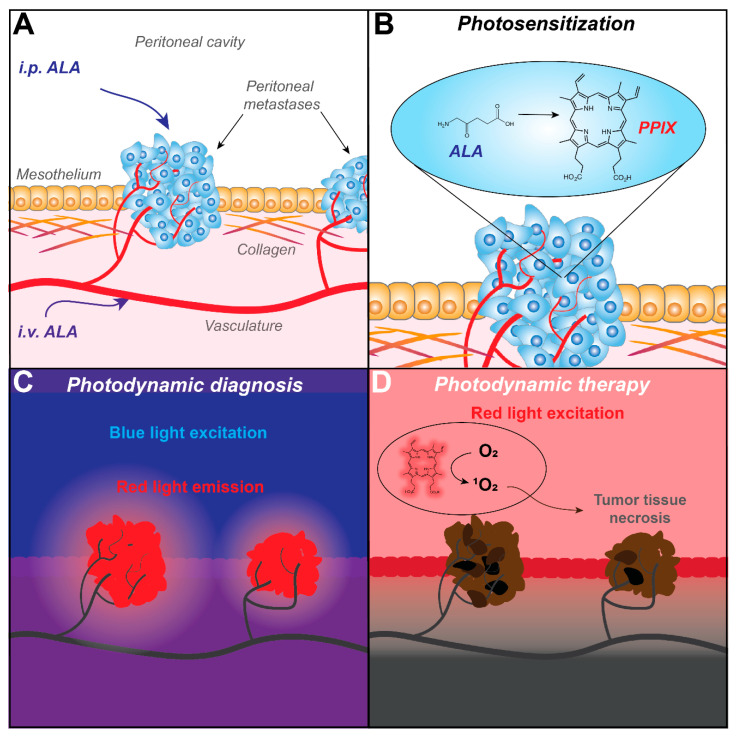
Fluorescence identification of peritoneal carcinomatosis (PCAR) by photodynamic diagnosis (PDD) and its subsequent treatment with photodynamic therapy (PDT), exemplified with ALA as a photosensitizer. (**A**) The photosensitizing agent ALA can be administered via either intraperitoneal (i.p.) or oral administration (i.v.) and accumulates in cancer tissues. (**B**) In cancer cells, ALA is preferentially metabolized to form PpIX, a fluorescent photosensitizer. (**C**) Under blue light, PpIX is excited and emits red fluorescence. Its detection can guide the surgical resection of the fluorescent tissues. (**D**) By exciting PpIX, for example with red light, reactive oxygen species such as ^1^O_2_ are formed through photochemical reactions and energy transfer. These cause oxidative damage to vital cellular components such as lipids, proteins and nucleic acids, culminating in massive tumor cell death.

As a diagnostic modality, PDD takes advantage of the fluorescence emission that can occur when light-excited photosensitizers relax from a singlet excited state back to their ground state. The principle of PDD has overlap with conventional fluorescence-guided surgery approaches, but distinguishes itself by using photosensitizers, i.e., agents that can also be used for PDT. Indeed, PDD has mostly been performed with 5-aminolevulinic acid (ALA) and its derivatives as photosensitizing agents. ALA is a rate-limiting precursor in the heme synthesis pathway, which is metabolized into the photosensitizer protoporphyrin IX (PpIX). Subsequently, ferrochelatase inserts ferrous iron into PpIX to form heme, which is non-fluorescent and has no photosensitizing properties. Although heme synthesis can occur in most cell types, exogenous ALA administration leads to PpIX buildup in cancer cells with remarkable specificity. Exogenous ALA is taken up by β-amino acid transporters that are typically upregulated in cancer cells, although this uptake mechanism may not apply to a clinically used methylated form of ALA [18]. In addition, the buildup of PpIX can be attributed to the elevated activity of the heme synthesis pathway in combination with the reduced activity of ferrochelatase, which normally catalyzes the final conversion of PpIX into heme [19]. PpIX is a potent photosensitizer that exhibits strong absorption around 409 nm (Soret band), and with lower efficiency at 505 nm, 540 nm, 575 nm and 630 nm (Q-bands). Upon excitation, fluorescence is emitted at 635 nm and 705 nm [20]. These fluorescent properties are highly useful for the identification of cancer tissues during surgery, and facilitate the accurate and more comprehensive fluorescence-guided resection of cancer tissues (Figure 1). An overview of clinically used and experimental photosensitizers for PDD of PCAR, which will be more elaborately discussed in this review, is given in Table 1.

As a therapeutic modality, PDT uses the ability of light-excited photosensitizers to produce high levels of reactive oxygen species. Light-excited photosensitizers have a strong tendency to undergo intersystem crossing from a short-lived singlet excited state to a longer-lived triplet excited state. From this triplet state, photosensitizers engage in photochemical reactions with molecular oxygen in order to decay back to their ground state. Depending on the photosensitizer, Type I or Type II photochemical reactions may occur, resulting in the generation of superoxide anion (O_2_^−^) or singlet oxygen (^1^O_2_), respectively [25] (Figure 1). When generated in cancer tissues, reactive oxygen species are highly cytotoxic as they cause wide-spread oxidative damage to proteins, lipids and nucleic acids [26]. In response, cancer cells can undergo various forms of cell death with apoptotic and necrotic phenotypes. Besides these direct cytotoxic effects, PDT also eradicates cancer tissues through potent secondary effects. Following systemic administration, photosensitizers accumulate in the vascular and perivascular areas of cancer tissues. Upon excitation by light, extensive cell death results in vascular collapse, thrombosis and blood flow stasis, which causes severe tumor hyponutrition and hypoxia [27]. Massive cell death also attracts neutrophils and dendritic cells, for which the recognition of tumor-specific antigens and oxidized proteins can stimulate a prolonged anti-tumor immune response [28]. In contrast to PDD, PDT has been performed with a large variety of photosensitizers, of which porfimer sodium (Photofrin), benzoporphyrin derivative (verteporfin/Visudyne), meso-tetrahydroxyphenylchlorin (mTHPC, temoporfin), padeliporfin (Tookad) and ALA (Levulan/Metvix) are among the most utilized. An overview of clinically used and experimental photosensitizers for PDT of PCAR, which will be more elaborately discussed in this review, is given in Table 1.

In this review, a brief overview of the clinical state-of-the-art and the major challenges of PDD and PDT in the management of PCAR are presented. Selected experimental studies are then highlighted, as their findings may provide new perspectives on addressing some of these challenges. This overview is intended to identify the remaining challenges, and to define further research into the highly promising application of PDD and PDT in the treatment of PCAR from varying primary cancer origins. 

## 2. Photodynamic Diagnosis for Peritoneal Carcinomatosis

### 2.1. Clinical State-of-the-Art

Compared to conventional cytoreductive surgery under white light, PDD presents a promising modality to improve the detection rate of micrometastases with high sensitivity and specificity [29,30,31,32,33,34,35,36,37,38,39,40]. However, despite these promising results, PDD remains an experimental diagnostic tool which is not yet widely applied. In this section, we present an overview of relevant clinical trials published since 2010 and discuss their major findings. Almost all trials used ALA, given 2–4 h preoperatively with an oral dosage ranging from 10 to 20 mg/kg [29,30,31,32,33,34,35,36,37,38,39,40]. Exposure to sunlight was avoided during 24 h after administration to prevent phototoxicity, and all studies report the absence of unacceptable morbidity. In 2015, Yonemura et al. reported grade 3–4 complication rates of 15.2% in patients with PCAR of various origins undergoing PDD-guided surgery [31]. In 2006, Sugarbaker et al. reported grade 4 morbidity in 19% of PCAR patients undergoing cytoreductive surgery and i.p. chemotherapy [41]. In 2017, Dhir et al. reported grade 3–4 morbidity in 26% of patients (aged 40–65 years) undergoing cytoreductive surgery and HIPEC [42]. The obvious difference is the absence of chemotherapy in the PDD study by Yonemura et al. In addition, these papers were not all published around the same time, so the advance in disease management may be another confounding factor. A fair evaluation of the morbidity of PDD-guided surgery compared to conventional cytoreductive surgery is thus still lacking. Nonetheless, the reported safety and feasibility studies of PDD have been highly encouraging.

#### 2.1.1. Ovarian Cancer

PDD is highly effective in the diagnosis of PCAR of ovarian cancer; PDD with a dose of 10 mg/kg ALA, orally administrated 4–9 h prior to cytoreductive surgery, achieved a sensitivity of 75% and specificity of 100%, without severe side-effects [38]. Higher doses of 20 mg/kg ALA, with a more convenient shorter incubation time of 2 h, have also been evaluated, reporting a sensitivity of 95% and specificity of 100% for ovarian PCAR with accurate detection of micrometastases [34]. Studies of Yonemura et al. compared the diagnostic performance of PDD for PCAR from different origins and illustrated that ALA-mediated PDD seemed to be more favorable for ovarian cancer, resulting in a sensitivity of nearly 90% and specificity of up to 100% in the presence of mild adverse effects in a small number of patients [35,37].

#### 2.1.2. Gastric Cancer

The application of ALA-guided PDD has also been explored for PCAR originating from gastric cancer. Kishi et al. focused on the staging laparoscopy for advanced gastric cancer either with or without prior treatment with 15–25 mg/kg of ALA, reporting an acceptable sensitivity of 80–90% and specificity ranging from 45% to 100% [29,32,36]. Despite the heterogeneity in specificity, the detection rate was still improved compared to the conventional observation under white light, represented by newly identified peritoneal metastases in at least 10% of the patients [29,32,36]. One study specifically reported that prior chemotherapy caused increased false positive detection rates, leading to a relatively lower specificity [36]. However, a smaller study in which a lower ALA dose of 10–15 mg/kg was used reported a sensitivity and specificity of 100% during the white light surgery in gastric cancer patients [30]. 

#### 2.1.3. Colorectal Cancer

ALA-PDD for PCAR of colorectal origin was shown to slightly enhance the diagnostic accuracy compared to observation under ambient light for patients that were suspected to have serosal invasion [33]. The effectiveness was determined at a sensitivity of 53% [37]. However, the auto-fluorescence in the surrounding tissues were believed to partially impair the sensitivity of PDD [33]. Further investigations are necessary to determine the nature of these unsatisfactory results, and suggest that efforts to enhance the sensitivity of PDD for this cancer type may be required.

#### 2.1.4. Pancreatic Cancer

One study reported on PDD in 34 patients of pancreatic cancer, in which oral ALA was given 3 h prior to cytoreductive surgery at a dose of 20 mg/kg. Suspicious lesions were identified under white light, were positively identified using PDD, and were then confirmed to be metastatic lesions, representing a sensitivity and specificity of 100% [40].

#### 2.1.5. Other Cancer Types

Besides secondary PCAR, ALA-mediated PDD is also a feasible tool for diagnosing primary peritoneal malignancies. PDD with ALA was used to detect primary peritoneal papillary serous carcinoma, wherein the diameter of the smallest nodule detected was 0.5 cm [43]. This was much larger compared to the aforementioned studies, and further exploration is warranted to determine the cause of this reduced accuracy. PDD with intravenous indocyanine green has been explored in patients with suspected metastases of hepatocellular carcinoma, achieving highly promising outcomes with a sensitivity and selectivity of 100% [31]. The major benefit of using indocyanine green compared to ALA/PPIX photosensitization is that its excitation and emission maxima lie at longer wavelengths, thus allowing the excitation light to penetrate deeper through tissues.

#### 2.1.6. Predictive Diagnosis with PDD

In addition to the increased detection rate, PDD also carries prognostic value that aids in designing treatment regimens. The peritoneal invasion status can be determined by ALA-mediated PDD to predict the prognosis of gastric cancer patients [39]. PDD can figure out much earlier the patients with occult peritoneal metastasis, enabling the early intervention with chemotherapy and improving overall patient survival [39]. Currently, no research has determined the relation between PDD efficacies and the recurrence rates.

### 2.2. PDD for Peritoneal Carcinomatosis: Challenges to Overcome

Despite providing satisfying results, these studies identified clear challenges regarding the use of PDD for PCAR. Most clinical studies were limited in terms of sample size and indicate a strong need for larger randomized and controlled clinical trials. Such clinical studies need to be homogenized in terms of design. For instance, most studies only selected ALA-negative suspicious nodules without healthy tissue controls, so that the predictive value might be over-estimated. Sometimes, not all fluorescent nodules were biopsied for pathological assessment. Besides, in some studies only patients with visible lesions under white light were re-diagnosed by PDD to verify and further explore the possible missed ones, while other patients with normal-like peritoneum were not selected to undergo PDD. In such cases, the accuracy of PDD was not optimally evaluated and positive lesions may have been missed.

#### 2.2.1. Tumor Heterogeneity

The heterogeneity of tumor origin and prior treatment may influence the sensitivity of PDD. Preoperative chemotherapy may lead to the degeneration of affected peritoneum, which can thus influence the uptake of ALA in residual lesions. However, chemotherapy prior to PDD has also been reported to increase the false positive detection rates due to inflammatory responses [36]. The inter- or intra-patient heterogeneity is another concern, as differences in gene expression patterns may influence the PpIX synthesis, and thus the ease with which they can be identified during PDD [35]. The biomarkers of PDD efficacy need to be investigated so as to better define the patients suitable for PDD, or to design biomodulation strategies to augment PpIX biosynthesis. 

#### 2.2.2. Increasing Specificity and Selectivity

Another challenge lies in increasing the accuracy of PDD, in particular for gastric- and colorectal PCAR, which requires the development of novel photosensitizers or molecular probes. False positive rates were shown to be related to hyperplasia caused by inflammation surrounding cancer tissues [36]. However, inflammation-associated fibrosis may result in false negative outcomes, as it may reduce the penetration depth of the blue excitation light and impair the ability of the emitted fluorescence to reach the detector [32]. Stromal tissues may also reduce the uptake of ALA by cancer cells, and thus reduce the efficacy with which metastases can be detected [35].

#### 2.2.3. Light Sources

The detection of PCAR by current illumination approaches also remains a challenge. The surface of peritoneum is rather large, and micrometastases may be optically shielded, which may prevent their detection. It has been reported that the therapeutic impact of fluorescence-guided surgery was limited by the performance of the optical detection device [44,45]. Furthermore, the treatment of the entire peritoneal cavity or persistent metastases of epithelial ovarian cancer in macroscopically healthy peritoneum was found to be intolerable [44]. As blue light has a limited penetration depth in tissue, the development of novel molecular or nanoscale probes that are excitable in (near) infrared regions may aid in improving PCAR detection. Taken together, advances in lighting solutions for intraperitoneal PDD, for improved tolerability and diagnostic properties, are strongly demanded [46,47]. 

### 2.3. Promising Experimental Studies on PDD for Peritoneal Carcinomatosis

**Figure 2:**
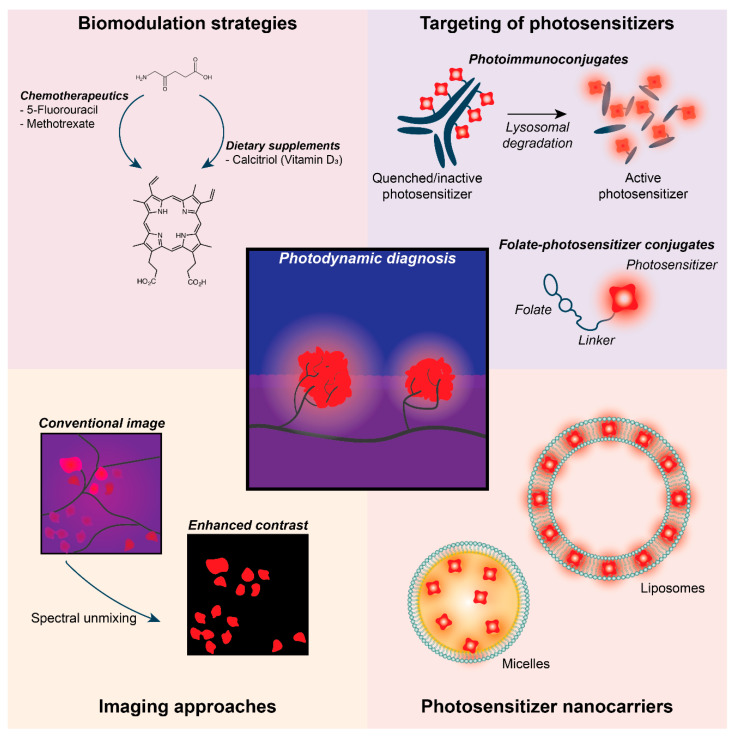
Promising strategies to improve the efficacy of PDD for PCAR. Innovative approaches have demonstrated the promise of biomodulation strategies in augmenting PpIX biosynthesis in cancer cells, in the targeting of photosensitizers, in the development of nanocarriers, and in improving imaging techniques.

Almost all studies performed with animal models of PCAR used PDD based on ALA (PpIX) [48], which has been shown to be safe and effective [44,48]. Fluorescence-guided surgery mediated by ALA-based PDD shows excellent accuracy, capable of detecting up to three times more peritoneal metastases than conventional white light laparoscopy [44,45,47,48,49,50]. ALA-PDD can also increase the detection of metastases that are 30 times smaller compared to detection with white light and the naked eye [44,45,48]. In this section, we will highlight various studies that may partially resolve some of the identified challenges of PDD for PCAR (Figure 2).

#### 2.3.1. Increasing Specificity and Selectivity 

Various biomodulation strategies have been developed to augment PpIX biosynthesis in cancer cells (Figure 2). In both models of human skin cancer, combinations of ALA and either methotrexate or calcitriol (vitamin D3) were shown to greatly amplify PpIX synthesis in vitro and in vivo. This resulted from upregulated coproporphyrinogen oxidase and decreased ferrochelatase protein expression [51,52,53]. A similar effect was reported in human skin cancer models when ALA was combined with 5-fluorouracil [54,55], a chemotherapeutic often used for the treatment of PCAR. Other biomodulation strategies involve the chelation of iron to reduce ferrochelatase activity, the enzyme responsible for inactivating the photosensitizing properties of PpIX by converting it to heme. The use of deferoxamine (Desferal), a commercially available and widely used chelator, was shown to be highly effective in increasing PpIX accumulation in cancer cells [56] and human skin fibroblasts [57]. Further studies are required to translate such combination therapies into a useful modality for PCAR.

To reduce false detection rates caused by inflammatory responses, the design of specific photosensitizers for PCAR that mitigate side effects on healthy tissue have been investigated [46]. Combining a molecular probe with a photosensitizer can enhance tumor targeting and the detection of lesions by PDD [46,50]. Bacteriochlorin-based photosensitizers conjugated to galactosyl human serum albumin, which binds lectin receptors on ovarian cancer cells, allowed the detection of peritoneal ovarian cancer metastases with very low false positives [58]. The specific fluorescent labeling of cancer cells with folate receptor-targeted fluorophores has been a widely adopted strategy in the clinical application of fluorescence-guided surgery for ovarian cancer [59,60,61], and similar strategies to conjugate photosensitizers to folate receptor-overexpression cells have been developed [62,63,64,65] (Figure 2). By exploiting the enhanced permeability and retention effect of cancer tissues, nanoscale drug carriers can be used for the selective accumulation of photosensitizers in peritoneal micrometastases. For instance, indocyanine green-loaded lactosomes accumulated well in the peritoneal micrometastases of gastric cancer cells in mice, whereas non-specific fluorescence was observed with the free dye [66]. 

Another innovative approach revolves around the use of photoimmunoconjugates: cancer-targeted antibodies onto which the photosensitizers are chemically conjugated (Figure 2). A clinical pilot study investigated the use of anti-carcinoembryonic antigen–fluorescein immunoconjugates in patients with colon cancer for photodiagnosis [67]. Ex vivo imaging of resected tumor specimens demonstrated a 10-fold higher fluorescence emission in cancer tissues compared to normal mucosa [67], demonstrating the clinical feasibility of photoimmunoconjugates for PDD. Another promising example is the use of cetuximab onto which self-quenching concentrations of benzoporphyrin derivative were conjugated. Following intraperitoneal injection in mice bearing OVCAR-5 ovarian cancer micrometases, these immunoconjugates were shown to bind the EGFR-overexpressing cancer cells. Subsequent receptor internalization and lysosomal degradation of the photoimmunoconjugates resulted in the unquenching of the photosensitizer, after which fluorescent detection and PDT could be achieved with enhanced selectivity and efficacy [68]. It should be noted that these photoimmunoconjugates have not yet been used for fluorescence-guided cytoreductive surgery. 

#### 2.3.2. Light Sources

Several advances have been made to achieve more accurate PDD imaging of PCAR. In comparison to blue light, near-infrared light penetrates deeper into tissues, which can result in higher PDD accuracy. On gastric cancer PCAR, using indocyanine green and 760 nm excitation, PDD provided good visualization and detection of the lesions, which was not possible with the naked eye [47,69]. By using PDD with real-time spectral unmixing to reduce background autofluorescence, a more accurate PDD of metastases was achieved in resected lymph nodes of colorectal cancer patients [70] (Figure 2). Rather than using lasers coupled to fiberoptic light distrubutors, light-emitting diodes offer more flexibility in reaching the target tissues as they can be assembled in different geometries [50]. Further improvements may be achieved by using specific wavelengths and light energies to prevent the photobleaching of the photosensitizers [48]. 

## 3. Photodynamic Therapy for Peritoneal Carcinomatosis

### 3.1. Clinical State-of-the-Art

Since the PCAR lesions can be easily accessed with optical fibers during either laparoscopic or open surgery (i.e., during cytoreductive surgery), PDT is a promising therapeutic option. In addition, since the PCAR lesions are typically small and superficial, the efficacy of PDT may be minimally affected by the limited penetration of light in tissue [20,71]. Though quite a few preclinical studies displayed inspiring results, there have only been a handful of clinical trials. The existing clinical trials have mostly been performed by one center between 1990 and 2006, with no new trial results published since 2012. These trials have almost exclusively been performed with intravenous porfimer sodium, a first-generation photosensitizer approved for clinical use in 1993. Patients received doses in the range of 1.5–3 mg/kg, and had to be shielded from light for up to 60 days post-treatment to avoid phototoxicity. In the following section, we will discuss the major findings per cancer type. 

#### 3.1.1. Safety and Feasibility Studies

In a phase I trial, the feasibility and the toxicity of porfimer sodium-PDT was investigated in patients with various forms of PCAR, including ovarian and gastro-intestinal cancers [72,73]. After evaluating the corresponding clinical outcomes, the maximal tolerated regimen was identified as 3.75 J/cm^2^, at 514 nm, targeting the whole peritoneal cavity, in addition to 5.0–7.5 J/cm^2^ (at 514 nm) or 10–15 J/cm^2^ (at 630 nm), targeting large lesions with 2.5 mg/kg porfimer sodium [73]. There existed a trade-off between the penetration depth and the risk of perforation of the gastro-intestinal tract. In terms of safety, PDT induced light dose-related pleural effusions, which was considered to be associated with the prolonged intubation and gastro-intestinal perforation [73]. After a median follow-up of 22 months, 78% of ovarian cancer patients had a median recurrence time of 4 months [73]. Since this concerned a Phase I safety and feasibility study, the survival benefit versus conventional treatments was not evaluated.

#### 3.1.2. Ovarian Cancer

With the use of 2.5 mg/kg porfimer sodium given intravenously two days prior to cytoreductive surgery, PCAR patients were primarily irradiated with light at 630 nm, but at 532 nm in cases where the mesentery and the bowel were targeted. Total radiant exposures ranged from 2.5 to 15 J/cm^2^, depending on the target sites [74,75,76]. Only a small number of patients could achieve a complete response, as measured 6 months post-operatively by the absence of abdominal disease under laparoscopy or medical imaging. Until the end of follow-up, all the patients relapsed with a median progression-free survival of 3 months and an overall survival of 22 months [74]. 

#### 3.1.3. Gastric/Intestinal Cancer

In the same study, the efficacy of the same PDT treatment parameters was evaluated in patients with gastro-intestinal cancers. Fewer patients went into complete remission as measured 6 months post-operatively [74]. The recurrence rate was 100%, with a progression-free survival of 3.3 months and overall survival of only 13 months [74], which is comparable to patients undergoing cytoreductive surgery and HIPEC [77]. The lower treatment response of gastric cancer patients was consistent with its poorer prognosis than ovarian cancer [7,8]. 

#### 3.1.4. Primary PCAR

Compared to secondary PCAR, the treatment response for malignant mesothelioma achieved more promising results. Chen et al. reported that the overall survival of patients undergoing a combination of cytoreductive surgery and porfimer sodium-PDT, with an initial dose of 200–250 J/cm^2^ followed by a second dose ranging from 0.5 to 1 times greater than the previous one, achieved a significantly improved median overall survival. Patients receiving a comprehensive treatment consisting of surgery, PDT and intraperitoneal cisplatin had a median overall survival of 64 months, whereas patients receiving only intraperitoneal cisplatin had a median overall survival of 9 months [78]. No severe complications were reported, and the much-relieved pain and improved performance status led to an improvement of quality of life [78]. 

#### 3.1.5. Adverse Events

Capillary leak syndrome characterized by fluid redistribution and hypovolemia was the most common PDT-related complication [74,75,76]. Other adverse events frequently involved mild photosensitivity, gastro-intestinal abnormalities and transient increases of transaminase levels [74,76]. Unlike the phase I trial, perforation of the gastro-intestinal tract was no longer a major concern due to improvements in surgical techniques [73].

#### 3.1.6. Ongoing Trials

A pilot Phase III study (NCT 02840331, University of Tübingen Medical Center, Tübingen, Germany) is ongoing, in which PDD and PDT with the photosensitizer hyperycin is being explored for patients with locally advanced gastric cancer. Though no results have been posted yet, this study may have guiding significance for future development, as a new generation photosensitizer is going to be employed.

### 3.2. PDT for Peritoneal Carcinomatosis: Challenges to Overcome

Based on these clinical studies and several recent reviews, the major challenges related to the use of PDT for the clinical management of PCAR can be identified. Regarding the design of clinical trials, these need to be standardized as much as possible, so that individual trials can be more easily compared. For each new photosensitizer with distinct optical properties, maximum tolerable photosensitizer doses, light doses and optimal drug–light time intervals need to be determined. Subsequent studies need to be homogenized regarding the type of primary cancer, the prior treatments, and the reported outcome parameters. 

#### 3.2.1. Tumor Heterogeneity

To explore the factors related to unsatisfactory treatment responses, the relation between porfimer sodium uptake and tissue oxygenation were studied. Although porfimer sodium uptake in PCAR of ovarian and gastric cancer was significantly higher compared to normal tissues, the differences were rather low [79]. Small tumor nodules had functional vasculatures, and were thus capable of porfimer sodium uptake [80]. Tumor nodules with normal oxygenation exhibited a much more heterogeneous uptake than more hypoxic lesions [81], which may be less susceptible to PDT as a consequence. 

#### 3.2.2. Selectivity and Efficacy of PDT

PDT with the reported photosensitizers has been associated with high morbidity, prolonged skin photosensitivity and limited therapeutic gain. These issues have been considered a major drawback of using PDT for PCAR. However, they mainly associate with the use of porfimer sodium, a first-generation photosensitizer with relatively low cancer selectivity, which exhibits prolonged accumulation in the skin. New photosensitizers have since been developed and have been approved for clinical use for different cancer types, which include ALA, liposomal verteporfin, temoporfin and padeliporfin. In addition to having lower adverse toxicities compared to porfimer sodium, they typically exhibit molar extinction coefficients that are 10–100-fold higher at clinically relevant wavelengths (600–700 nm), thus enabling more effective absorption of the excitation light. Further studies are required to investigate whether these photosensitizers have sufficient tumor specificity and efficacy for the treatment of PCAR. 

To further address this issue and reduce PDT-associated morbidity, nanocarriers and (immune-) targeting for photosensitizers may need to be developed for increased specificity for cancer tissues. Nanoscale drug carriers, such as micelles or liposomes, may extravasate specifically at the cancer site through the well-described enhanced permeability and retention effect [82,83]. Active targeting can further enhance the selective uptake of either molecular or nanocarrier-bound photosensitizers [84,85,86,87]. Moreover, active targeting may be explored for the intraperitoneal administration of photosensitizers, which could significantly reduce the systemic causes of PDT-associated morbidity [45,88]. Although some candidate epitopes for molecular targeting have been identified for ovarian cancer [89], more studies are required to identify suitable targets for PCAR of colorectal, pancreatic and gastric origins. For ALA, biomodulation strategies to increase endogenous PpIX production as described for PDD may have similar relevance to augmenting the efficacy of ALA-PDT. 

#### 3.2.3. Integration into Clinical Practice

Approaches for the implementation of novel photosensitizers and excitation sources need to be compatible with the relevant clinical practice, i.e., cytoreductive surgery combined with chemotherapy. Regarding cytoreductive (open) surgery, the application of intraoperative or immediate post-operative PDT appears feasible. As such, PDT may be used to sterilize the resected areas of micrometastases or dislodged cancer cells to reduce recurrence rates. Moreover, the effects of PDT are required to not affect the efficacy of chemotherapeutics. Some promising headway has been made regarding PDT–chemotherapy combinations for PCAR, which will be further discussed below.

#### 3.2.4. Excitation Sources

Lastly, it remains challenging to resolve the limited coverage and penetration of the excitation light throughout the peritoneal cavity, making the destruction of occult nodules hard. Inventive means of intraperitoneal light irradiation are necessary, and various new approaches have been investigated, as further discussed below. 

### 3.3. Promising Experimental Studies on PDT for Peritoneal Carcinomatosis

**Figure 3:**
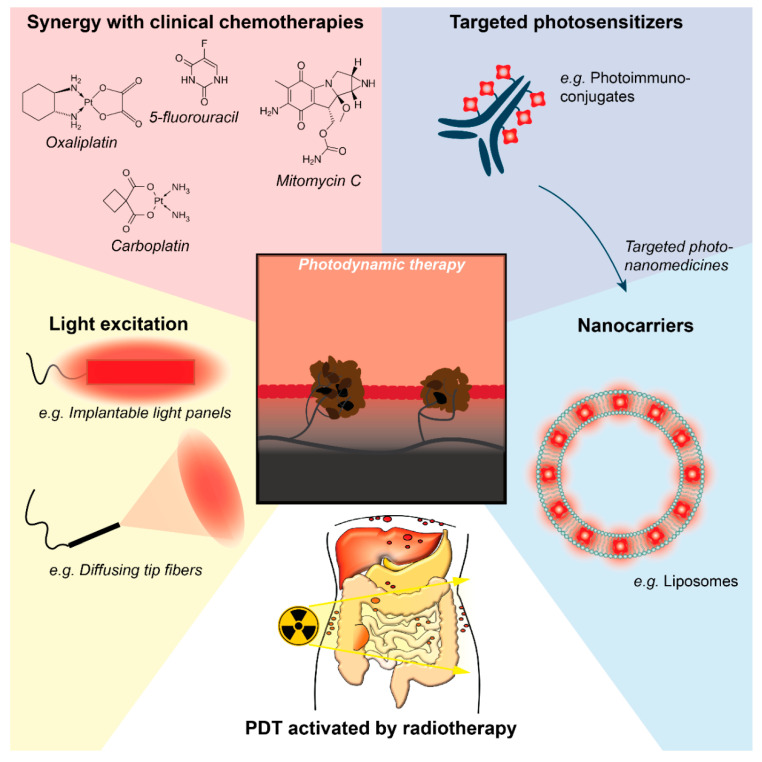
Promising strategies to improve the efficacy of PDT for PCAR. Promising results from the application of PDT for PCAR have been achieved using innovative photoimmunoconjugates and photosensitizer-loaded nanocarriers, which can in principle be combined to develop targeted photonanomedicines. Regarding the integration into the clinical practice, PDT may synergize with clinical chemotherapies, and innovative lighting solutions (e.g., implantable light panels and diffusing tip laser wands) may aid in improving the efficacy of PDT for PCAR. To overcome the difficulty of reaching occult PCAR with light, radiotherapy-activated PDT may be a promising solution.

Although PDT has seen limited clinical success, various preclinical studies have demonstrated promising results with diverse photosensitizers [88,90]. In the section below, we highlight several promising studies in which some of the identified challenges were tackled through the development of targeted PDT strategies, combinations of PDT with clinically-used chemotherapeutics, and innovative photosensitizer excitation methods (Figure 3).

#### 3.3.1. Selectivity and Efficacy of PDT

Various studies have achieved promising results on the selectivity and efficacy of PDT for PCAR. We already highlighted the work of Spring et al. on the development of photoimmunoconjugates composed of cetuximab, onto which self-quenching concentrations of verteporfin were conjugated. When intraperitoneally injected into nude mice carrying ovarian PCAR, these immunoconjugates achieved tumor-to-normal ratios in the range of 9–18, which was significantly higher than free verteporfin (2.7) [68]. In this study, intraperitoneal PDT was performed by injecting an intralipid solution into the peritoneal cavity for optimal light dispersion, followed by 690 nm irradiation using a diffuse tip fiber that was consecutively aimed at the different peritoneal quadrants. While the maximum tolerated PDT dose for free verteporfin (0.25 mg/kg) was 8 J/cm^2^, the maximum tolerated PDT dose for the photoimmunoconjugates (2 mg/kg verteporfin) was between 50–100 J/cm^2^. No notable morbidity or mortality was ascribed to PDT with these photoimmunoconjugates, indicating improved selectivity compared to non-targeted PDT protocols. The authors further report an 89% reduction in the micrometastatic burden in PDT treated animals compared to non-treated animals [68], although no further survival studies were performed. 

To selectively target photosensitizers to cancer cells, folate receptor targeted photosensitizers have been developed, as this receptor is overexpressed by a majority of ovarian cancer cells [62]. The photosensitizer pyropheophorbide conjugated to folate was demonstrated to be effective in binding ovarian cancer cells in a rat model of ovarian PCAR, with a reported tumor-to-normal ratio of 9.6 [62]. Follow-up studies showed the effective PDT of ovarian and pancreatic cancer cell lines in vitro using this folate-conjugated photosensitizer [89,91], indicating that this targeting strategy may have relevance for pancreatic PCAR as well.

A recent study by our group showed that the encapsulation of verteporfin in nanostructured lipid carriers allowed cellular uptake and high phototoxicity when exposed to 690 nm laser light in ovarian cancer cells cultured in monolayers and in 3D-spheroids [92]. When injected intravenously into mice with ovarian tumors or into an orthotopic mouse model of human ovarian PCAR, these nanoparticles demonstrated a long circulation time associated with efficient tumor uptake (tumor-to-normal ratio of 11.5), including in peritoneal small tumor nodules. Furthermore, PDT with 690 nm laser light exposure (200 J/cm^2^), 24 h after the intravenous administration of verteporfin-loaded nanoparticles (8 mg/kg verteporfin), significantly inhibited tumor growth without visible toxicity [92]. In contrast, free verteporfin (2 mg/kg) combined with 50 J/cm^2^ laser light exposure induced severe phototoxic adverse effects. The lipid nanoparticles thus led to efficient verteporfin vectorization to the tumor site and protection from photosensitizer systemic adverse effects, providing promising therapeutic prospects for PDT in ovarian cancer and PCAR in combination with conventional surgery.

#### 3.3.2. Integration into Clinical Practice

A highly promising approach is the integration of PDD and PDT using one active agent. This strategy has been explored on rat models of ovarian PCAR with ALA-based PDD under blue light, followed by ALA-based PDT with 630 nm (5–20 J/cm^2^) to kill the cancer nodules. This was even followed up with ALA-based PDD to assess the efficacy of the procedure [93,94]. Although the procedure was feasible and effective, the low selectivity of intraperitoneal ALA administration was associated with treatment-induced mortality [93,94]. With the emergence of novel targeted photosensitizers and photoimmunoconjugates to improve the tolerability of PDT, as described above, the combination of PDD-guided surgery and PDT may hold significant promise in the management of PCAR.

With respect to the effect of PDT on clinically used adjuvant chemotherapeutics, various studies have typically reported beneficial outcomes for such combination treatments. With the use of 3D microtumor models of micrometastatic ovarian cancer, a combination of verteporfin-PDT followed by carboplatin chemotherapy was demonstrated to be synergistic. These findings were attributed to the capacity of PDT to disrupt the microtumor’s integrity, allowing carboplatin to penetrate deeper into the tissues [95]. In vivo, PDT with adjuvant aspirin may also aid in improving drug extravasation; a study on the chorioallantoic membrane model demonstrated that while PDT induced vascular occlusion, PDT combined with aspirin could delay thrombosis and enhanced the permeability of the treated blood vessels [96]. Such approaches could benefit combination therapies of PDT and chemotherapies by improving the availability of therapeutics through the typically dense cancer microenvironments. 

With the use of the aforementioned cetuximab-based photoimmunoconjugates, PDT combined with a paclitaxel-cisplatin regimen was also shown to yield beneficial outcomes in an in vivo model of ovarian cancer PCAR [68]. In 3D cultures of micrometastatic pancreatic cancer, verteporfin-PDT was also shown to augment the efficacy of oxaliplatin [97]. Combinations of low-dose mitomycin C, 5-fluorouracil, doxorubicin, cisplatin and vincristine were also shown to act synergistically with PDT on gastric cancer cell lines (primary cancer origin) [98]. For primary colorectal cancer, synergies of PDT with mitomycin C have been reported, using in vitro and in vivo models [99,100]. Thus, although no complete overview of combinations of PDT and clinically used chemotherapies for the different PCAR subtypes is available, these findings are highly encouraging when considering the compatibility of PDT with the current clinical practice of PCAR management. 

#### 3.3.3. Light Sources

To improve the efficacy of the excitation light in reaching cancer tissues, Guyon et al. described the use of a light irradiation panel that could be implanted intraperitoneally, and which achieved homogeneous illumination of the peritoneal cavity [101]. The same group has developed various light-emitting fabrics for the treatment of dermal lesions [102], and adaptations of such approaches may hold promise for light delivery in PCAR. As previously discussed, the use of intralipid is also a promising method of intraperitoneal light dispersion with endoscopic light irradiation [68,73]. A study on malignant pleural mesothelioma described the application of a spherical diffusing fiber placed within an inflatable silicone balloon to excite tumor-localized, PEGylated mTHPC [103]. The authors reported uniform light distribution throughout the thoracic cavity, and the protection of photothermal toxicity in tissues that were in contact with the balloon [103]. Such approaches may hold promise for intraperitoneal light delivery. Although the use of light will always be met with a limited penetration depth in tissue, alternative ways to excite photosensitizers need to be considered [71], and exciting novel approaches will be discussed further in Section 3.4.

### 3.4. Radiotherapy-Activated PDT for PCAR

**Figure 4:**
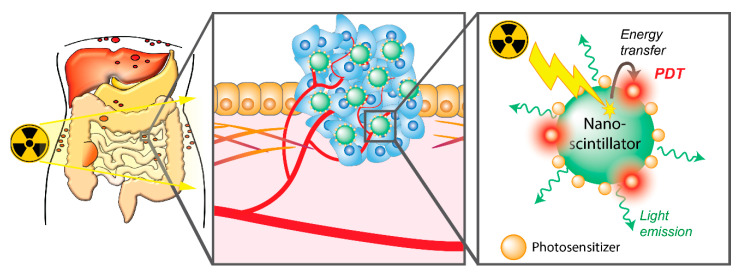
Schematic overview of the use of radiotherapy-activated PDT for PCAR using radioluminescent nanomaterials. Radiotherapy can be applied for whole-abdomen irradiation. Scintillating nanomaterials accumulated in the cancer tissues can absorb the X-ray radiation and down-convert it into visible light. Conjugated photosensitizers can be excited, resulting in the photodynamic production of cytotoxic reactive oxygen species.

Although PCAR lesions are superficial and the limited penetration of light is not a major limiting factor for the treatment of well-identified and accessible metastases, a major challenge lies in the treatment of undetectable microscopic disease or tumors that are optically shielded by organs and that strongly affect the overall survival of the patients. Secondly, it is difficult for light to homogeneously excite photosensitizers throughout the whole abdominal cavity. To tackle these challenges, a novel approach to induce PDT by X-ray radiation has recently emerged. Because tissues are nearly transparent to X-rays, this strategy enables the remote excitation of photosensitizers, in a non-invasive way and potentially within the entire peritoneal cavity during the course of radiotherapy (Figure 4). This technology relies on exciting nanoparticles, which are capable of down-converting ionizing radiations, such as X-rays, into visible light [104]. When conjugated to photosensitizers, nanoscintillators can convert the X-rays used in radiotherapy into visible light that can subsequently excite the photosensitizers. Encouraging results demonstrating the ability of this X-PDT strategy have been obtained in vitro and in vivo, which have been recently reviewed [105,106,107,108,109,110].

One important consideration is whether radiotherapy would fit in the treatment regimen for PCAR. With respect to ovarian cancer, radiotherapy is regaining interest as regards the treatment of PCAR. While radiotherapy was once included in the standard-of-care following cytoreductive surgery, it was gradually replaced by emergent chemotherapy regimens that exhibited better outcomes. Indeed, in addition to inducing acute and long-term toxicity, radiation therapy has lacked efficiency in handling the disease [111,112]. Retrospective studies demonstrated that radiotherapy efficacy was impaired by a non-complete irradiation of the abdominal cavity, and whole abdominal cavity irradiation has since been investigated. With whole abdominal cavity radiation, radiotherapy demonstrates good results by lengthening both the relapse-free survival and overall survival [113,114,115,116]. In combination with carboplatin, radiotherapy achieved 45% relapse-free survival at 5 years versus 19% for chemotherapy alone, and a 5-year survival rate of 59% versus 26% for chemotherapy alone [114]. To indicate the used dose, this study delivered a total of 51.6 Gy to the pelvic area in 1.8 Gy fractions, whereas 42.6 Gy were delivered to the para-aortic region in fractions of 1.5 to 1.8 Gy. Subsequently, a large randomized clinical trial was performed in patients diagnosed with advanced metastatic ovarian cancer. In this trial, whole abdominal radiotherapy was compared to chemotherapy as an adjuvant strategy, following cytoreductive surgery and an induction chemotherapy. While no significant difference was obtained between whole irradiation radiotherapy and chemotherapy in patients with residual microscopic disease, the 5-year progression-free survival was significantly enhanced after radiotherapy (56%) versus chemotherapy (36%) when patients had a complete surgical and pathological remission after surgery [117]. 

To overcome the toxicity associated with the irradiation of organs at risk, recent technological developments can be leveraged to deliver radiotherapy in a more accurate matter. For instance, intensity-modulated radiotherapy has been shown to efficiently spare the organs at risk while delivering a homogeneous irradiation dose throughout the abdominal cavity [118]. A multicenter phase II clinical trial demonstrated that intensity-modulated radiation therapy is indeed a valuable strategy to safely deliver homogeneous irradiation to the whole abdominal cavity, with a tolerable toxicity profile and no impairment of the quality of life. The study was performed on 20 patients with optimally debulked peritoneally metastasized ovarian cancer that achieved complete remission after carboplatin/paclitaxel chemotherapy. The patients received a total dose of 30 Gy, delivered in 20 fractions of 1.5 Gy each [119,120]. By strongly decreasing the toxicity generally associated with whole abdominal irradiation, this strategy thus presents whole abdominal radiotherapy as a new adjuvant therapy for ovarian cancer PCAR [121,122,123]. Taken together, state-of-the-art protocols can achieve homogeneous radiation dose delivery throughout the entire peritoneal cavity with acceptable acute and long-term toxicity.

Although further randomized clinical trials are necessary to confirm the encouraging trends obtained for the progression-free survival and overall survival, whole cavity irradiation appears to be a safe and feasible option for ovarian cancer patients with PCAR. As such, radiotherapy can be leveraged for X-PDT, in which tumor-localized nanoscintillator-photosensitizer conjugates combined with whole abdominal cavity irradiation may achieve PDT throughout the entire peritoneum in a minimally invasive and homogeneous manner. Further research is necessary to evaluate the efficacy of this innovative approach, to determine whether such strategies would also work for PCAR of alternate origins, and whether additional targeting strategies are required to achieve satisfactory treatment outcomes. 

## 4. Conclusions and Perspectives

PDD and PDT are promising techniques to address the difficulties in the diagnosis and treatment of PCAR. Indeed, PDD has emerged as a valuable diagnostic tool, capable of improving the accuracy with which micrometastases can be identified and resected during surgery. However, PDT has thus far failed to achieve similar clinical success, despite the highly encouraging findings of various preclinical studies. The problematic tumor selectivity, as a major causal factor of these disappointing clinical results, has been well identified, and ongoing efforts in evaluating novel/alternative photosensitizers, the design of (immune) targeted approaches and the development of nanoconstructs will be crucial to overcome this challenge. Focused preclinical studies and/or clinical trials on the most promising photosensitization strategies may thus provide a better insight into the clinical value of PDT for the treatment of PCAR. 

The first unresolved challenge of both PDD and PDT relates to tumor heterogeneity. PCAR microtumors developing in different niches of the peritoneal cavity may have distinct phenotypes and metabolic preferences, which may influence their responses to PDD and PDT [124,125,126,127]. This may be worsened by selection pressures of adjuvant chemotherapeutics, inflammatory responses and treatment-induced fibrosis. Research is needed to globally characterize this heterogeneity, to correlate this to the PDD and PDT efficacies, and to identify strategies to address these potential issues. Reported biomodulation strategies that have been developed for skin and pancreatic cancer may improve the efficacy of PDD and PDT by reducing intratumor heterogeneity [52,54,124,128], but further investigations are required to determine whether similar treatments could address the varying responses of PCAR microtumors with distinct phenotypes. Such studies may be best performed on patient-derived in vitro and in vivo models, as these best recapitulate the intra- and inter-patient heterogeneity of PCAR.

A second unresolved challenge relates to the use of light, which is intrinsically limited in terms of penetration into tissue and in reaching optically occluded micrometastases. The use of radiotherapy to activate PDT may be a highly promising approach to overcome this challenge, as it penetrates deeply into tissue and can activate PDT as a non-invasive procedure. However, this innovative approach has never been developed for PCAR, and needs to be further investigated in terms of efficacy and clinical feasibility. This may be envisioned as a post-operative round of low-dose whole-abdomen radiotherapy, of which the compatibility with clinical chemotherapeutics and (PDD-guided) cytoreductive surgery needs to be determined. 

In conclusion, PDD holds significant potential in the management of PCAR by improving the accuracy and extensiveness of cytoreductive surgery. Large randomized and controlled clinical trials are needed to generate more compelling evidence for this approach, and promote its integration into the clinical practice. PDT is also a promising approach, although it is met with more serious challenges. As various innovative approaches regarding light delivery and higher-specificity photosensitization strategies are under development, the most restrictive challenges may be addressed. The clinical feasibility of PDT is also hopeful, as the currently-available evidence indicates that PDT exerts beneficial effects on clinically relevant chemotherapeutics for PCAR. Combined approaches of PDD-guided cytoreductive surgery and PDT may be especially promising in the short- to medium-term. In the long-term, X-ray-activated PDT may overcome the final limitations regarding the treatment of optically undetected metastases.

## Figures and Tables

**Table 1:** An overview of clinically used and experimental photosensitizers for PDD and PDT of PCAR.

Photosensitizer	Application	Peak Excitation Wavelengths	Molar Extinction Coefficient (M^−1^cm^−1^)	Peak Emission Wavelength	State of Development	Ref.
Aminolevulinic acid (PpIX)	PDD & PDT	409 nm 630 nm	1.2 × 10^5^ (409 nm) 5.0 × 10^3^ (630 nm)	635 nm	Clinical trials	[21]
Indocyanine green	PDD	780 nm	2.6 × 10^5^	835 nm	Clinical trials	[22]
Porfimer sodium	PDT	630 nm	1.2 × 10^3^	635 nm	Clinical trials	[21]
Hypericin	PDD & PDT	589 nm	4.5 × 10^4^	599 nm	Clinical trials	[21]
Pyropheophorbide A (Folate-conjugated)	PDD & PDT	668 nm	4.5 × 10^4^	672 nm	Preclinical	[23]
Meso-tetrahydroxy-phenylchlorin (Folate-conjugated)	PDD & PDT	652 nm	2.9 × 10^4^	655 nm	Preclinical	[21]
Benzoporphyrin derivative (anti-EGFR mAb-conjugated)	PDD & PDT	692 nm	3.3 × 10^4^	695 nm	Preclinical	[24]
